# Dissemination of atopic dermatitis and food allergy information to pregnant women in an online childbirth preparation class

**DOI:** 10.1016/j.jacig.2021.12.004

**Published:** 2021-12-29

**Authors:** Yusuke Inuzuka, Kiwako Yamamoto-Hanada, Rina Akaishi, Megumi Haruna, Manami Matsubara, Mayako Saito-Abe, Haruhiko Sago, Yukihiro Ohya

**Affiliations:** a Allergy Center, National Center for Child Health and Development, Tokyo, Japan; b Division of Obstetrics, Center for Maternal-Fetal, Neonatal and Reproductive Medicine, National Center for Child Health and Development, Tokyo, Japan; c Department of Midwifery and Women’s Health, Division of Health Sciences and Nursing, Graduate School of Medicine, The University of Tokyo, Tokyo, Japan

## Abstract

**Background:**

Evidence-based allergy prevention strategies have been reported, but strategies for dissemination have not been evaluated. Improving health literacy and awareness of allergies in pregnant mothers is 1 example of dissemination and implementation science that could help prevent allergic diseases and promote early detection of allergic diseases in children.

**Objective:**

We evaluated the usefulness of an online childbirth preparation class about prevention and early detection of allergic diseases in offspring.

**Methods:**

From January 2021 to August 2021, an online allergy class for pregnant mothers was provided at the hospital in Tokyo. We conducted an online survey about allergy topics before and after the online childbirth preparation class.

**Results:**

A total of 106 pregnant women attended the online allergy class, and 92 (86.8%) responded to the online survey. Of the respondents, 90 (97.8%) were worried about the development of allergies in their children. The topic that attracted the most attention in the lecture was the prevention of atopic dermatitis by means of skin care. The percentages of correct responses regarding allergy prevention strategies increased after the class. All mothers believed that the class was useful, the information should be disseminated to the public, and the practices should be implemented.

**Conclusion:**

In online childbirth preparation classes, information about allergy based on dissemination and implementation science could strengthen allergy literacy among pregnant women.

## Introduction

Allergies such as atopic dermatitis (AD) and food allergy are found worldwide. According to a nationwide birth cohort study in Japan, almost half of pregnant women and their partners have a history of allergic disease^1^; therefore, many babies are born with a genetically high risk for allergy. A previous study in Japan demonstrated that most mothers are worried about whether their offspring will develop allergic disease in the future, and the results of a survey raised concerns about allergy prevention.^2^ In randomized controlled trials, our study group previously confirmed that early skin care intervention in young children at high risk for AD could prevent the development of AD^3^ and that early introduction of peanut and hen’s eggs in the diet could also prevent the onset of peanut and egg allergy in young infants^4^^,^^5^; unfortunately, 44% of caregivers believed that egg introduction should be delayed in young children.^2^ Evidence-based allergy prevention strategies have been reported but not yet presented to the general public.

Dissemination and implementation (D&I) science is the development and verification of strategies for the effective and efficient incorporation of evidence-based interventions into health care activities. We believe that improving health literacy and awareness of allergies in pregnant mothers is 1 example of D&I science that could help prevent food allergies and other allergic diseases and promote early detection of allergic diseases in children. In this study, we evaluated the usefulness of an online childbirth preparation class about prevention and early detection of allergic diseases, especially AD and food allergy in offspring.

We conducted an online survey about allergy topics before and after the online childbirth preparation class. Such classes, which provide general information about pregnancy and infant care, are available at almost all maternity clinics in Japan. Our hospital, the National Center for Child Health and Development (NCCHD) in Tokyo, Japan, also has a general childbirth preparation class. From January 2021 to August 2021, an additional online allergy class for pregnant mothers was provided at the NCCHD. The participants were pregnant women who had seen posters and leaflets about our study and wished to participate. This class was a 1-hour online lecture (delivered through the Microsoft Teams platform) presented by a pediatric allergist, who provided information about various allergies described in the online supporting information. The content of the allergy class was developed by pediatricians, allergists, obstetrics and gynecology specialists, and midwives (all coauthors). Survey responses from before and after the class were compared by using a chi-square test or Fisher exact test, performed by using EZR, version 1.30 (R Foundation for Statistical Computing, Vienna, Austria). The NCCHD institutional review board approved the study. All participants provided informed consent to publish the findings.

## Results and discussion

A total of 106 pregnant women attended the online allergy class, and 92 of them (86.8%) responded to the survey. Patient characteristics are listed in Table E1 (in the supporting information, which is available at www.jaci-global.org). The median age of the participants was 35.5 years (interquartile interval = 32.0-38.0), and 72 of the participants (78.3%) were primiparas. The educational level of the participants was high, with 83 (90.2%) having an undergraduate or graduate degree. Of the respondents, 66 (71.7%) had allergic diseases, and 90 (97.8%) were worried about the development of allergies in their children. The most concerning allergic disease was AD. The topic that attracted the most attention in the lecture was the prevention of AD by skin care. The percentages of correct responses regarding allergy prevention strategies increased after the class (Table I). All of the mothers believed that the class was useful, the information should be disseminated to the public, and the practices should be implemented. The participants provided many comments. These included the following: “I was very anxious, but after taking this lecture, I felt the need to respond correctly. The more I searched on the Internet, the less I knew what to do, so it was conducive.” and “I learned that the thorough discussion, the information on the Internet was incorrect, and I was able to learn evidence-based content, which was a great learning experience.”

**Table I:** Responses to all survey statements before and after the lecture (N = 92)

Survey statements/responses	Correct answer	No. of participants (%)		*P* value
		Before lecture	After lecture	
Q1. Eliminating foods that cause a high frequency of allergic reactions during pregnancy can prevent allergies in newborns.	No			
Yes		1 (1.1)	0 (0.0)	.00
No		70 (76.1)	91 (98.9)	
I do not know		20 (21.7)	1 (1.1)	
Q2. Removing foods that frequently cause allergies while the mother is breast-feeding will prevent allergies in the child.	No			
Yes		4 (4.3)	0 (0.0)	.00
No		58 (63.0)	91 (98.9)	
I do not know		30 (32.6)	1 (1.1)	
Q3. Children are also prone to allergic diseases if their parents have allergic diseases.	Yes			
Yes		75 (81.5)	84 (91.3)	.08
No		4 (4.3)	2 (2.2)	
I do not know		13 (14.1)	6 (6.5)	
Q4. Topical steroids are not recommended for children's skin, particularly the skin of those with eczema.	No			
Yes		12 (13.0)	3 (3.3)	.00
No		51 (55.4)	88 (95.7)	
I do not know		29 (31.5)	1 (1.1)	
Q5. Complete breast milk is recommended to prevent milk allergies in the absence of artificial milk.	No			
Yes		8 (8.7)	2 (2.2)	.00
No		55 (59.8)	82 (89.1)	
I do not know		29 (31.5)	8 (8.7)	
Q6. It is better to start baby food later in infancy to prevent food allergies.	No			
Yes		2 (2.2)	0 (0.0)	.00
No		64 (69.6)	91 (98.9)	
I do not know		26 (28.3)	1 (1.1)	
Q7. It is better to start eating eggs later in infancy to prevent egg allergies.	No			
Yes		7 (7.6)	0 (0.0)	.00
No		54 (58.7)	91 (98.9)	
I do not know		31 (33.7)	1 (1.1)	

In the preclass survey, many of the women expressed the desire for accurate, evidence-based information on childhood allergies and prevention thereof.

This study demonstrated that the inclusion of allergy information in online childbirth preparation classes could strengthen allergy literacy among pregnant mothers. To our knowledge, this is the first evaluation of online allergy education classes for pregnant mothers.

Appropriate evidence-based information could help improve quality of life and reduce parents’ worries about allergy onset in children. According to previous studies, poor mental health in pregnant women was linked to maternal allergy^6^ and could promote the postnatal development of food allergy and asthma in their children.^7^ Traditional childbirth preparation classes do not generally include information about pediatric allergy. We propose that the content of such classes be updated to include current pediatric health issues and maternal mental health. In this study, classes were delivered online because of the coronavirus disease 2019 (COVID-19) pandemic, but online lectures may be preferable for many mothers even after the COVID-19 pandemic because of the accessibility of the class.

There were several limitations to this study. First, it was not a randomized controlled trial with a control group for comparison. Second, there may have been a selection bias in this study. Because participants were self-selected, they may be more interested in allergies than the general population. Furthermore, the educational level of the pregnant women was high. In the future, we would like to provide such a class for a broader demographic of pregnant women with various education levels to evaluate its usefulness. Third, we evaluated only knowledge and feelings about allergies before and after the class. We were unable to evaluate the implementation of preventive strategies. We hope to conduct further research into this in the future. Moreover, the effect of different educational modalities—online, in person, or via smartphone—and the optimal timing of these interventions for the improvement of health literacy and outcomes should also be considered in further research. Education of families in real-time during postnatal health visits may be optimal for the dissemination of information about allergy management and prevention strategies. Families with poor health literacy may benefit from other educational formats such as videos rather than traditional didactic modalities. These have the advantage of being rewatchable during the first months of the infant's life, thus increasing the chance that they may alter parental behaviors. We would like to modify our next study with these points in mind. Fourth, the sample size of this study was small. We are planning a larger study to evaluate the feasibility and efficacy of this intervention.

The concept of evidence-based medicine has greatly influenced medical care and public health activities, and evidence-based guidelines have been established. However, interventions whose effectiveness has been proved in randomized controlled clinical trials are not always promptly adopted and routinely implemented in real-world diagnosis, treatment, health care, and public health.^8^ We hope that inclusion of useful allergy information based on D&I science in an online childbirth preparation class will be implemented extensively and will improve quality of life and allergy outcomes in children and their families.

## Supplementary data

Supplementary Material

Table E1
