# Supplementary material for: Dissemination of atopic dermatitis and food allergy information to pregnant women in an online childbirth preparation class
Source: J Allergy Clin Immunol Glob. 2021 Dec 29;1(1):24–6. doi: 10.1016/j.jacig.2021.12.004 (PMC10509843; doi:10.1016/j.jacig.2021.12.004)
Supplement: Supplementary Material [file mmc1.docx]

Online Repository File

Lecture content of online class

- What is an allergy?
- What is an allergic march?
- Atopic dermatitis
- Diagnostic criteria
- Description of the disease
- Treatment
- Steroid ointment
- Exacerbation factor
- Food allergy
- Description of the disease
- Immunoglobulin E antibody
- Dual allergen exposure hypothesis
- Asthma
- Description of the disease
- Prevention of allergic diseases
- Dietary restrictions during pregnancy and lactation
- Prevention of atopic dermatitis by skin care after birth
- Starting baby food
- Relationship between the period from the appearance of eczema to treatment and food allergy
- Q and A
- Are allergic diseases inherited?
- Relationship between pets and allergies
- Relationship between baby swimming and asthma
- About probiotics
- Milk allergy and breast milk/artificial milk
