# Supplementary material for: Dissemination of atopic dermatitis and food allergy information to pregnant women in an online childbirth preparation class
Source: J Allergy Clin Immunol Glob. 2021 Dec 29;1(1):24–6. doi: 10.1016/j.jacig.2021.12.004 (PMC10509843; doi:10.1016/j.jacig.2021.12.004)
Supplement: Table E1 [file mmc2.docx]

Table E1 Participant characteristics

| Characteristic (N = 92) | | Data |
| --- | --- | --- |
| Median age (years) |  | 35.5 (IQR, 32–38) |
| Primipara (*n*) |  | 72 (78.3%) |
| Final educational background (*n*) | Graduate school | 16 (17.4%) |
|  | University | 67 (72.8%) |
|  | Junior college | 4 (4.3%) |
|  | Vocational school | 4 (4.3%) |
|  | High school | 1 (1.1%) |
| Maternal history of allergies (*n*) | Atopic dermatitis | 28 (30.4%) |
|  | Food allergy | 11 (12.0%) |
|  | Asthma | 12 (13.0%) |
|  | Allergic rhinitis | 51 (55.4%) |
|  | Urticaria | 6 (6.5%) |
|  | Drug allergy | 5 (5.4%) |
| I am worried that my newborn will have an allergic disease. | |  |
| Strongly agree (*n*) |  | 27 (29.3%) |
| Agree (*n*) |  | 63 (68.5%) |
| Not agree (*n*) |  | 2 (2.2%) |
| I have gathered information to prevent my child from suffering from allergic diseases. | |  |
| Yes (*n*) |  | 35 (38.0%) |
| No (*n*) |  | 57 (62.0%) |
| I have done something to prevent my child from suffering allergic diseases. | |  |
| Yes (*n*) |  | 7 (7.6%) |
| No (*n*) |  | 85 (92.4%) |

IQR, interquartile range.
